# The Role of Self-Perceptions of Aging for Recovery of Older Adults After a Hospital Stay

**DOI:** 10.1093/geroni/igaa057.1970

**Published:** 2020-12-16

**Authors:** Anne Blawert, Ellen Freiberger, Susanne Wurm

**Affiliations:** 1 University Medicine Greifswald, Greifswald, Mecklenburg-Vorpommern, Germany; 2 Institute of Biomedicine of Aging, Friedrich-Alexander-University Erlangen-Nurnberg, Nurnberg, Bayern, Germany

## Abstract

For older adults, a hospital stay can lead to loss of physical function and frailty. It is therefore important to investigate factors for recovery after hospitalization. Recent studies suggest negative self-perceptions of aging (SPA) as a potential risk factor in the context of serious health events. This ongoing longitudinal study investigates how negative SPA might contribute to worse physical recovery (assessed with the Short Physical Performance Battery) after hospital stay in a sample of 244 German adults aged 75 to 96. Preliminary mediation analysis based on available data of the first 50 participants indicate that negative SPA is related to increased fear of falling after 6 months, which predicts worse physical function one year after hospitalization (indirect effect: B = -0.70, SE = 0.41, p = .09). The results stress the importance of SPA for health recovery in old age and introduce fear of falling as a psychological pathway.

